# Kleine-Levin Syndrome- an Exceedingly Rare Recurrent Hypersomnia Disorder in an Adolescent Male: A Case Report

**DOI:** 10.1192/bjo.2026.11781

**Published:** 2026-06-30

**Authors:** Tumpa Ghose

**Affiliations:** Black Country Health Care NHS Foundation Trust, Birmingham, United Kingdom

## Abstract

**Aims:**

This case reports aim to describe the clinical presentation, diagnostic evaluation, and treatment response of an adolescent male with Kleine-Levin Syndrome, thereby increasing clinical awareness and contributing to the limited evidence on effective management strategies for this rare condition.

**Methods:**

A 16-year-old adolescent male with a one-year history of recurrent episodes of hyperphagia, excessive sleepiness, hypersexuality, cognitive impairment, and behavioural disturbances was evaluated. Each episode lasted approximately one week and recurred every 2-3 months, with complete inter episodic recovery. Detailed clinical history and comprehensive physical, neurological, and psychiatric examinations were performed. Laboratory investigations, karyotyping, brain magnetic response imaging, and electroencephalography were conducted to exclude alternative neurological, metabolic, and psychiatric diagnoses. The diagnosis of Kleine-Levin syndrome was established based on clinical features and fulfilment of the International Classification of Sleep Disorders, Third Edition (ICSD-3) criteria. Pharmacological treatment with methylphenidate and carbamazepine was initiated, and the patient was followed up at two-month intervals to assess treatment response.

**Results:**

Baseline investigations, including laboratory tests, MRI, and EEG, were unremarkable. Following treatment initiation, the patient demonstrated significant clinical improvement. The duration of hypersomnia episodes decreased from approximately seven days to three to four days, and episode frequency reduced from every 2-3 months to every 4-5 months. The severity of associated hypersexual behaviour, cognitive disturbances, and irritability also diminished. Inter episodic functioning and overall quality of life improved, with no reported adverse effects from treatment.

**Conclusion:**

This case highlights the importance of considering Kleine-Levin syndrome in adolescents presenting with recurrent episodic hypersomnia and behavioural changes. Early diagnosis and appropriate pharmacological intervention may reduce symptom severity and episode frequency. Further research is required to better understand the pathophysiology and establish standardized treatment guidelines for KLS.

